# Efficacy and safety of administering oral misoprostol by titration compared to vaginal misoprostol and dinoprostone for cervical ripening and induction of labour: study protocol for a randomised clinical trial

**DOI:** 10.1186/s12884-018-2132-3

**Published:** 2019-01-08

**Authors:** O. Lapuente-Ocamica, L. Ugarte, A. Lopez-Picado, F. Sanchez-Refoyo, Iñaki Lete Lasa, O. Echevarria, J. Álvarez-Sala, A. Fariñas, I. Bilbao, L. Barbero, J. Vicarregui, R. Hernanz Chaves, D. Paz Corral, J. A. Lopez-Lopez

**Affiliations:** 1 Department of Obstetrics and Gynecology, Araba University Hospital, Jose Atxotegui s/n, 01009 Vitoria-Gasteiz, Spain; 2 Bioaraba Research Unit, Jose Atxotegui s/n, 01009 Vitoria-Gasteiz, Spain; 3 Araba Research Unit, University Hospital Araba, c/ Jose Atxotegui s/n, 01009 Vitoria-Gasteiz, Spain; 4 Clinical Research and Clinical Trials Unit, Hospital Clínico San Carlos. Instituto de Investigacion Sanitaria del Hospital Clínico San Carlos (IdISSC), C/ Profesor Martin Lagos s/n, 28040 Madrid, Spain; 5 Department of Pediatrics, Araba University Hospital, Jose Atxotegui s/n, 01009 Vitoria-Gasteiz, Spain; 6 Pharmacy Department, Araba University Hospital, Jose Atxotegui s/n, 01009 Vitoria-Gasteiz, Spain

**Keywords:** Induction, Labour, Misoprostol, Dinoprostone, Efficacy, Safety

## Abstract

**Background:**

Among the various methods available, the administration of prostaglandins is the most effective for inducing labour in women with an unfavourable cervix. Recent studies have compared treatment with various titrated doses of oral misoprostol with vaginal misoprostol or dinoprostone, indicating that the use of an escalating dose of an oral misoprostol solution is associated with a lower rate of caesarean sections and a better safety profile. The objective of this study is to assess which of these three therapeutic options (oral or vaginal misoprostol or vaginal dinoprostone) achieves the highest rate of vaginal delivery within the first 24 h of drug administration.

**Methods:**

An open-label randomised controlled trial will be conducted in Araba University Hospital (Spain). Women at ≥41 weeks of pregnancy requiring elective induction of labour who meet the selection criteria will be randomly allocated to one of three groups: 1) vaginal dinoprostone (delivered via a controlled-release vaginal insert containing 10 mg of dinoprostone, for up to 24 h); 2) vaginal misoprostol (25 μg of vaginal misoprostol every 4 h up to a maximum of 24 h); and 3) oral misoprostol (titrated doses of 20 to 60 μg of misoprostol following a 3 h on + 1 h off regimen up to a maximum of 24 h). Both intention-to-treat analysis and per-protocol analysis will be performed.

**Discussion:**

The proposed study seeks to gather evidence on which of these three therapeutic options achieves the highest rate of vaginal delivery with the best safety profile, to enable obstetricians to use the most effective and safe option for their patients.

**Trial registration:**

NCT02902653 Available at: https://clinicaltrials.gov/show/NCT02902653 (7th September 2016).

**Electronic supplementary material:**

The online version of this article (10.1186/s12884-018-2132-3) contains supplementary material, which is available to authorized users.

## Background

Currently, there are pharmacological (administration of prostaglandins or isosorbide mononitrate) and mechanical (insertion of balloon catheters or cervical dilators) methods for cervical ripening [1, 2]. Prostaglandins are considered the most effective agent in women with an unfavourable cervix [3], these molecules being effective for both cervical ripening and the induction of labour [2, 4–6]. Their main adverse effect is excess uterine activity with or without cardiotocographic abnormalities [1, 2, 4, 6], and these effects are route of administration and dose dependent [1].

### Vaginal dinoprostone

Dinoprostone, a synthetic analogue of prostaglandin E2, is available in various formulations, including intracervical gel, controlled-release vaginal systems, and vaginal tablets. The controlled-release vaginal system available in our health system is an insert containing 10 mg of dinoprostone with a release rate of 0.3 mg/h that can be left in the vagina for up to 24 h. An advantage of this system is that in the event of uterine tachysystole or cardiotocographic abnormalities, the device can be removed easily. Compared to placebo, vaginal dinoprostone is associated with a higher rate of vaginal delivery within the first 24 h of drug administration without decreasing the rate of caesarean sections, but also with a higher risk of uterine hyperstimulation with changes in foetal heart rate (FHR) [2].

### Misoprostol

Misoprostol, a synthetic analogue of prostaglandin E1, is indicated for the prevention and treatment of peptic ulcer disease [7]. This drug stimulates the endometrium, induces uterine contractions and is effective for cervical ripening [1–4, 8]. In addition, its low cost and the fact that can be stored at room temperature make it a key drug for the induction of labour in developing countries [9, 10]. Misoprostol can be administered by various routes: buccal, oral, rectal, sublingual and vaginal [1, 11]. The dose and duration of administration of this drug, however, have not yet been well defined [12, 13].

### Vaginal misoprostol

With vaginal administration, the concentration of misoprostol reaches a peak plasma concentration after 70 to 80 min and then decreases, levels remaining detectable up to 6 h post-administration [14]. The vaginal administration of a 50-μg dose of misoprostol has been widely studied, results indicating that this dose has a greater efficacy but is less safe than a 25-μg dose (being associated with higher rates of uterine tachysystole, uterine hyperstimulation, and caesarean section due to an abnormal cardiotocographic findings and meconium) [15, 16]. Although the World Health Organization recommends using 25 μg every 6 h, several studies have assessed the administration of this dose every 5 h, finding no increase in abnormal events [17–19] and a higher rate of vaginal delivery within 12 h of starting induction than with dinoprostone [11].

A 2010 Cochrane review comparing vaginal misoprostol with placebo, vaginal and intracervical dinoprostone and oxytocin showed that vaginal misoprostol at doses > 25 μg every 4 h is more effective than the other conventional methods for induction of labour, but with a higher rate of uterine hyperstimulation, while low doses of this drug (≤25 μg every 4 h) were found to have the same levels of effectiveness and risk as other methods of inducing labour [16]. In line with this, another systematic review concluded that vaginal misoprostol is more effective than vaginal dinoprostone and they have similar safety profiles [5].

Subsequently, in 2011, Silfeler et al. conducted a study comparing vaginal misoprostol (25 μg every 4 h up to a maximum of 8 doses), controlled-release vaginal dinoprostone (10 mg over 24 h) and oxytocin in women with intact membranes [18]. They observed that misoprostol was more effective than dinoprostone or oxytocin, achieving a rate of vaginal delivery of 48.5% within 12 h in the misoprostol group compared to 36.1 and 13.3% in the oxytocin and dinoprostone groups, respectively. No significant differences were found in terms of uterine hyperstimulation rate or neonatal outcomes.

More recently, in 2014, Abraham et al. carried out a retrospective study on the induction of women with premature rupture of membranes using 25 μg vaginal misoprostol every 4 h up to a maximum of 6 doses vs 10 mg of dinoprostone over 12 h [17]. They concluded that vaginal misoprostol is more effective than dinoprostone for induction of labour in this population, without increasing the rate of adverse outcomes. Further, they found a caesarean section rate of 20% in the dinoprostone group compared to 11% in the misoprostol group [17].

### Oral misoprostol

Oral administration of misoprostol in solution is a route that is generally better tolerated by women since it involves fewer vaginal examinations [20]. After oral administration, misoprostol has a very short half-life of between 20 and 40 min [12]; the plasma concentration reaches an optimal level 30 min after administration and then decreases, the drug being cleared from the blood by 120 min [12, 14].

A 2011 review concluded that oral misoprostol is associated with a lower rate of caesarean sections than vaginal dinoprostone or placebo [2], and also with less uterine hyperstimulation than vaginal administration, without alterations in FHR. A 2014 Cochrane review [21] found a similar level of efficacy with oral and vaginal routes of administration but reported better perinatal outcomes with the oral route, and therefore recommended oral over vaginal administration.

Nevertheless, it is unknown what is the optimal regimen. Different studies have suggested different regimes and doses with successful but highly varied results, the rate of women achieving a vaginal birth within the 24 h of drug administration ranging from 54 to 94%. To date, it seems that titrated doses of misoprostol solution achieves better outcomes than fixed doses, as this produces constant blood levels of the drug [12].

In a clinical trial, Cheng et al. [22] compared the use of vaginal misoprostol (25 μg every 4 h) with a titrated oral misoprostol solution. The initial dose of misoprostol solution was 20 μg per hour, and this was increased by 20 μg every 4 h to a maximum of 60 μg. The rate of vaginal delivery within the first 24 h of drug administration was 94.1% with the oral route of administration, compared to 53.8% with the vaginal route, with no cases of uterine hyperstimulation. On the other hand, Rouzi et al. [12] reported a higher rate of vaginal delivery among women who received oral misoprostol (70% with misoprostol vs 55% with dinoprostone) and a lower rate of caesarean sections, but the difference did not reach significance. Finally, two recently published meta-analyses [10, 23] indicate that although vaginal misoprostol (≥50 μg) is associated with a higher success rate of vaginal delivery within the first 24 h of drug administration, a low dose of titrated oral misoprostol (< 50 μg) is associated with a lower caesarean section rate.

Since induction of labour is an increasingly widely used procedure, it is important to identify a pharmacological agent that is effective as well as safe. Given previous research findings, we expect in this open-label randomised clinical trial that the use of oral misoprostol may help improve the rate of vaginal labour while maintaining a good safety profile.

## Methods/design

### Aim

The objective of this study is to assess which of the three options considered, vaginal dinoprostone or vaginal or oral misoprostol, achieves a higher rate of vaginal delivery within the first 24 h after drug administration in women who undergo elective induction of labour due to their pregnancy becoming prolonged.

### Participants

This single-centre open-label randomised clinical trial will be carried out in Araba University Hospital (Spain), a tertiary hospital that conducts approximately 2600 deliveries per year. The study population consists of pregnant women at 41 weeks of pregnancy or more undergoing induction of labour who meet all the inclusion criteria and none of the exclusion criteria.

- Inclusion criteria: Women are required to be over 18 years old with a singleton pregnancy, cephalic presentation, intact membranes, a Bishop score of less than 6, and a cardiotocographic trace with a foetal heart rate pattern that is reactive with no decelerations, and provide written informed consent.
- Exclusion criteria: To be eligible, women must not have an allergy or intolerance to any of the study drugs; a history of caesarean section or uterine surgery, or of stillbirth; high parity (four or more previous births); contraindications for vaginal birth, including placenta or vasa praevia; intrauterine growth restriction; gestational hypertension; suspected chorioamnionitis; or blood clotting disorders, epilepsy, liver or kidney disease, or moderate-to-severe heart disease.

### Procedure

**Fig. 1:**
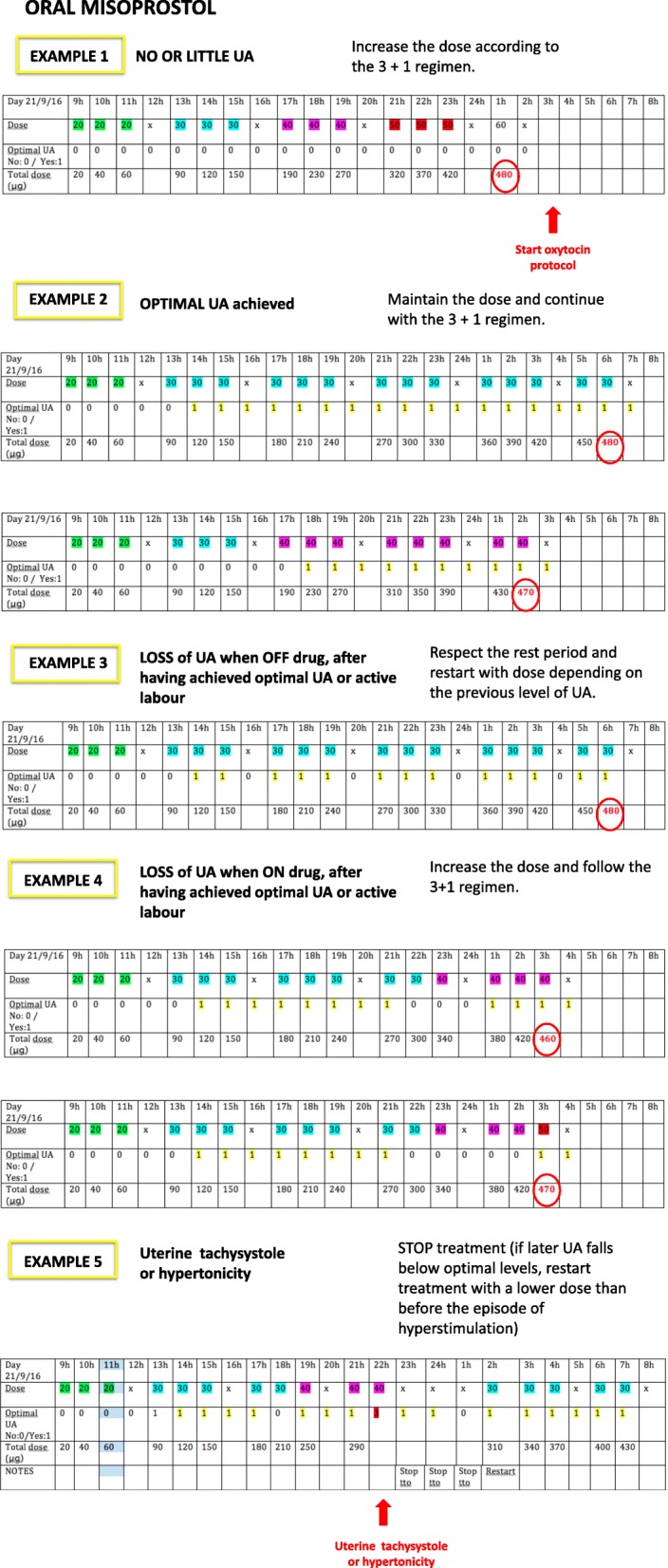
Schedule of enrollment, interventions, and assessments of the study

On the day of the induction attempt, having confirmed that women meet the selection criteria, they will be randomly allocated to one of three groups:

1. **Dinoprostone group:** women assigned to this group will have a controlled-release non-biodegradable polymer vaginal insert containing 10 mg of dinoprostone (Propess® FERRING Laboratories-Switzerland) inserted, and the insert will be left in the patient’s vagina for a maximum of 24 h.
2. **Vaginal misoprostol group (Misoprostol**_**v**_**):** women in this group will receive 25 μg of misoprostol (Misofar® BIAL laboratories, Spain) vaginally every 4 h for a maximum of 24 h (i.e., up to a maximum dose of 150 μg).
3. **Oral misoprostol group (Misoprostol**_**o**_**):** women in this group will be treated using a dose escalation design described in detail in Fig. 1 of this protocol. In brief, they will follow what we call a 3 + 1 regimen, corresponding to a 3-h period on the drug followed by 1 h off the drug (3 doses, one per hour + 1 h of rest).

At first, women will be given 20 μg of misoprostol orally every hour until adequate uterine activity is achieved (3 contractions every 10 min). If after 3 h of treatment (a total of 3 doses), uterine activity is not adequate, the drug will not be administered in the following hour, and after that 1 h of rest, one 30-μg dose will be administered each hour until adequate uterine activity is achieved, up to a maximum of three doses. If at this stage, there is still an inadequate uterine response, the same procedure will be followed, first with 40-μg doses and then with 50-μg doses, always allowing a 1-h rest period before increasing the dose. After another 1 h off, one last dose of 60 μg will be given, attempting to achieve adequate uterine activity. Once this 60-μg dose has been given, the treatment will be stopped, even if adequate uterine activity has not been achieved, and hence, in cases of inadequate uterine response, the maximum duration of this treatment would be 18 h.

In the event that women achieve adequate uterine activity at any point during the procedure, the latest dose will continue to be administered following the 3 + 1 pattern, that is, for every 3 h on the treatment, there will be 1 h off, up to a treatment duration of 24 h. Women assigned to this group will receive up to a maximum of 480 μg of oral misoprostol.

To summarise, the maximum duration of the intervention in the dinoprostone and vaginal misoprostol arms will be 24 h. In the oral misoprostol group, the intervention will also last for 24 h provided that the maximum dose is not reached, but will be stopped earlier if this dose is reached. Tables 1 and 2 summarise the treatment as a function of the progress of the induction process. Figure 1 includes some examples of the treatment. Additional files 1 and 2 include SPIRIT figure and checklist.

**Table 1:** Misoprostol dose escalation design

Scenarios	Oral Misoprostol	Vaginal Misoprostol	Vaginal Dinoprostone
No or little uterine activity (UA)	Increase dose according to the regimen: 3 h on + 1 h off (Example 1)	Maintain regimen	Maintain regimen
Optimal UA	Maintain the dose and continue with the regimen (3 h on + 1 h off) (Example 2)	Maintain regimen	Maintain regimen
Active labour	Maintain the dose and continue with the regimen (3 h on + 1 h off) (Example 2)	Maintain regimen	Maintain regimen
Loss of UA after optimal UA	- **When “off” drug:** respect the period of rest and restart with dose depending on the previous level of UA (following the 3 + 1 regimen) (Example 3) - **When “on” drug**: increase the dose and follow the 3 + 1 regimen (Example 4)	Maintain regimen	Confirm the presence of the drug in vagina:
			- Lack of drug: administer additional dose. - Presence: maintain regimen.
Active labour + loss of UA after optimal UA		When in combination with a lack of progression of labour, STOP treatment + oxytocin protocol	
Active labour + no progress	STOP treatment + oxytocin protocol		
Non-reassuring cardiotocographic pattern	STOP treatment + oxytocin protocol		
Uterine tachysystole or hypertonicity	STOP treatment (if later UA falls below optimal levels, restart treatment with a lower dose than before the episode of hyperstimulation) (Example 5)	STOP treatment (if later UA falls below optimal levels, restart treatment)	STOP treatment (if later UA falls below optimal levels, start oxytocin protocol)
End of treatment without UA	Oxytocin protocol		
End of treatment with UA	Watchful waiting + oxytocin protocol if loss of UA		

Optimal UA: at least 3 contractions lasting more than 60 s every 10 min

Little UA: less than 3 contractions every 10 min

Active labour: at least 4 cm of dilatation with optimal UA

No progress of labour: The following criteria must be met:

- Latent phase of labour completed and active phase of labour started (cervical dilation of 4 cm or more)

- Contraction pattern of 3 contractions every 10 min with adequate intensity for 4 h without cervical changes

Non-reassuring cardiotocographic trace:

- Recurrent late decelerations lasting for 30 min or more

- Atypical variable decelerations in more than 50% of the contractions for 30 min or more

- Prolonged decelerations: decrease in foetal heart rate (FHR) by ≥15 beats per minute (bpm) for 2 to 10 min

- Foetal bradycardia: FHR < 100 bpm for more than 10 min

- Reduction in variability indicating a need for intervention

- Sinusoidal FHR pattern

Uterine tachysystole: Six or more contractions in 10 min for at least 30 min

Hypertonicity: Sustained uterine contractions for more than 2 min without complete uterine relaxation

Uterine hyperstimulation: Excessive uterine activity with abnormal FHR

**Table 2:**
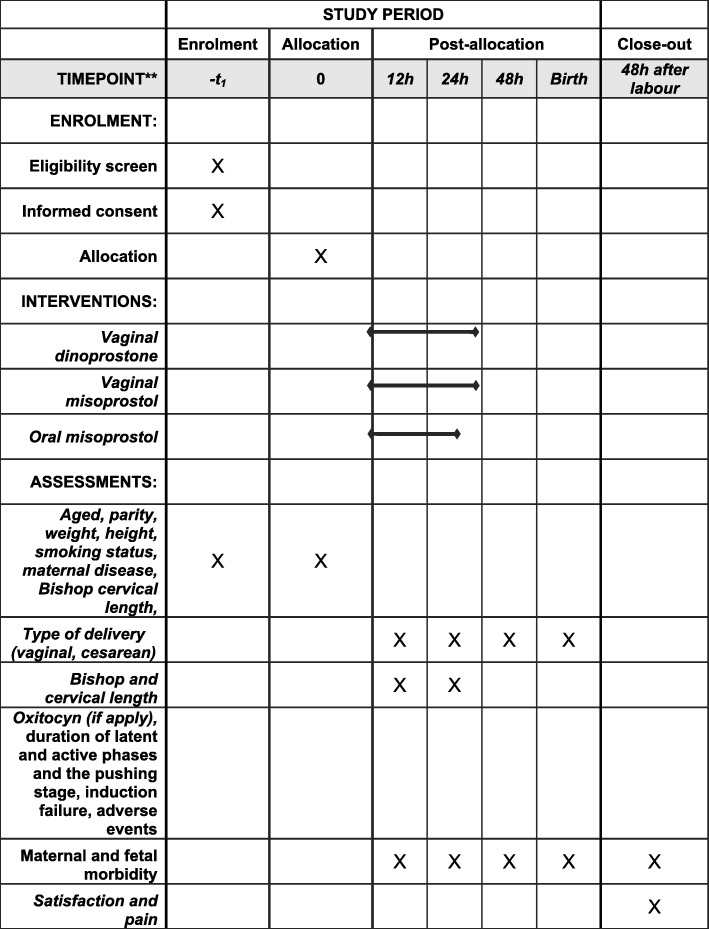
Schedule of enrolment, interventions, and assessments during the clinical trial

Additionally, in all of the groups, in the event that the maximum treatment period is completed without adequate uterine response, women will start to be given oxytocin for a maximum period of 24 h. If delivery is achieved by the end of this period, the induction will be considered a success. If induction is unsuccessful, a caesarean section will be performed.

Oxytocin infusion protocol: First, 10 units of oxytocin will be added to 500 ml of saline. The rate of infusion will be set at 3 ml/hour, doubling the dose every 20 min up to 24 ml/hour (yielding doses of 3, 6, 12 and 24 ml/h). Subsequently, the dose will be increased by 6 ml/hour every 20 min up to 120 ml/h. If uterine activity is still inadequate with this dose, 20 units of oxytocin in 500 ml of saline will be used with a starting rate of 60 ml/hour increasing the dose by 3 ml/hour every 20 min up to a dose of 90 ml/hour.

The women and their infants will be followed-up until discharge from hospital.

### Randomisation

Selected women will be randomly assigned in a 1:1:1 ratio to the dinoprostone, oral misoprostol or vaginal misoprostol groups using a computer-generated random number sequence. Researchers will be blinded to this randomisation sequence throughout the study. The randomisation will be performed through an online platform that is available 24-h a day and meets data protection requirements.

### Blinding

Given the characteristics of the intervention, we will not attempt to blind patients or clinicians.

### Sample size calculation

Taking data in the scientific literature on the rate of vaginal delivery within 24 h after the administration of the study drugs (70% for oral misoprostol [12], 55% for dinoprostone [12] and 54% of vaginal misoprostol [24]) as a reference, we will need to analyse 333 women (111 per arm) to detect significant differences between the groups with a power of 90% and a confidence level of 95%. Assuming a 10% rate of losses during the follow-up period, we will therefore need to recruit a total of 372 women (124 per arm).

### Variables

The data collected will be recorded on case report forms, safeguarding patient confidentiality at all times. The primary outcome will be the rate of vaginal delivery (normal or instrumental) within the first 24 h after drug administration.

The secondary outcomes include cervical status (Bishop score of 6 or over, assessed 12 and 24 h after drug administration), percentage of caesarean sections, use of oxytocin and, if so, the total dose, duration of latent and active phases and the pushing stage of labour, induction failure (no progress into the active phase of labour after administering oxytocin for 12 ± 3 h after membrane rupture), uterine tachysystole, hypertonicity, hyperstimulation, or rupture, adverse events including maternal and/or foetal death, pain (assessed on a visual analogue scale), women’s satisfaction with the induction process (assessed using a dichotomous question, asked 2 days after childbirth or on hospital discharge), maternal morbidity (uterine rupture, severe postpartum haemorrhage or intensive care unit admission) and foetal morbidity (1- and 5-min APGAR scores, umbilical cord pH, and neonatal asphyxia, among other variables).

Due to the extensive information about misoprostol and dinoprostone in the literature, it was considered that a data monitoring committee was not needed. We report to the ethics committees yearly on the progress of the trial. Currently, we have no plans to make any major modifications to the protocol, but we will communicate any such modifications to all people involved.

### Statistical analysis

First, we will describe the general characteristics of the sample. For this, percentages with 95% confidence intervals will be calculated for qualitative data and means and standard deviations for quantitative data.

Second, logistic regression analysis will be used to assess the relationship between the intervention received and the primary outcome (vaginal delivery within the first 24 h after administration of the corresponding drug). Crude and adjusted models will be constructed using variables found to be confounding factors in the univariate analysis.

Logistic regression analysis will also be used to analyse the rates of vaginal deliveries at 12 and 48 h after starting induction and the ripening of the cervix at 12 and 24 h. The rates of maternal adverse events (gastrointestinal adverse effects, uterine tachysystole, uterine rupture, uterine hyperstimulation, morbidity, postpartum haemorrhage, and fever) and of caesarean sections will be compared between the groups with a chi-square test.

Third, multiple linear regression analysis will be performed to explore the relationships between duration of the labour (total, and of active and latent phases), the maximum level of pain reported by the woman, 1- and 5-min APGAR scores, umbilical cord pH, and the intervention (vaginal dinoprostone, and oral or vaginal misoprostol). As well as crude models, adjusted models will be built controlling for intervention group. Complications and adverse events including neonatal asphyxia, morbidity, neonatal intensive care unit admission, and mortality and other complications will be compared between groups with a chi-square test.

Both intention-to-treat and per-protocol analysis will be performed. For the intention-to-treat analysis, we will consider all women included in the study who received at least one dose of any medicine. To handle any missing data on satisfaction, we will use multiple imputation techniques, taking into account age, parity and other potentially relevant variables. We will use the IBM SPSS Statistics for Windows, version 22.0, considering a *p* value of < 0.05 to be significant.

### Ethical considerations

Before inclusion, women will be asked to give written informed consent by their gynaecologist. This study has been approved by the Clinical Research Ethics Committee of Araba University Hospital and the Spanish Agency of Medicines and Medical Devices.

## Discussion

Given that a growing percentage of women undergo induction of labour [4, 5], it is important to determine the best method for this process. There are reasons to assert that misoprostol is the best drug, since as well as being inexpensive, it can be stored at room temperature, and has high efficacy and a good safety profile, especially when administered orally [25, 26].

On the one hand, this study is one of the few that attempts to use titrated doses according to uterine response, which may result in a lower rate of hyperstimulation and caesarean sections compared to vaginal administration of misoprostol [25]. Further, despite the safety associated with the rapid clearance of oral misoprostol [27], in our trial, we will progressively increase the doses (by 10 μg) with 1 h of observation off the drug for every 3 h in which doses are given, thereby further increasing the levels of safety.

On the other hand, our formulation allows more accurate dosing than those used in other studies [12, 25, 28] In previous studies, the drug in solution has been obtained by dissolving a 200-μg tablet of misoprostol in 200 ml of water, and the solution has been kept for up to 24 h. A syringe has been used to obtain the volumes to be given the women from this solution, assuming that in 1 ml of solution there will be 1 μg of drug [12, 28] This approach does not, however, take into account the potential precipitation of the drug or that it may not be homogenously dissolved, and hence, may lead to dosing errors. In contrast, in our study, we will use 20 and 30 μg capsules, which will be dissolved in water just before administration.

If the hypothesis of this study is confirmed, oral misoprostol in titrated doses may come to be considered the most effective and safe option for pre-induction/induction of labour in women with unfavourable cervix.

## Additional files

Additional file 1: SPIRIT figure. (PPTX 332 kb)

Additional file 2: SPIRIT checklist. (DOC 123 kb)

## Abbreviations

CRF: Case report form
FHR: Foetal heart rate
