# Supplementary material for: Efficacy and safety of administering oral misoprostol by titration compared to vaginal misoprostol and dinoprostone for cervical ripening and induction of labour: study protocol for a randomised clinical trial
Source: BMC Pregnancy Childbirth. 2019 Jan 8;19:14. doi: 10.1186/s12884-018-2132-3 (PMC6325751; doi:10.1186/s12884-018-2132-3)
Supplement: Supplementary file 1 — SPIRIT figure. (PPTX 332 kb) [file 12884_2018_2132_MOESM1_ESM.pptx]

## Slide 1
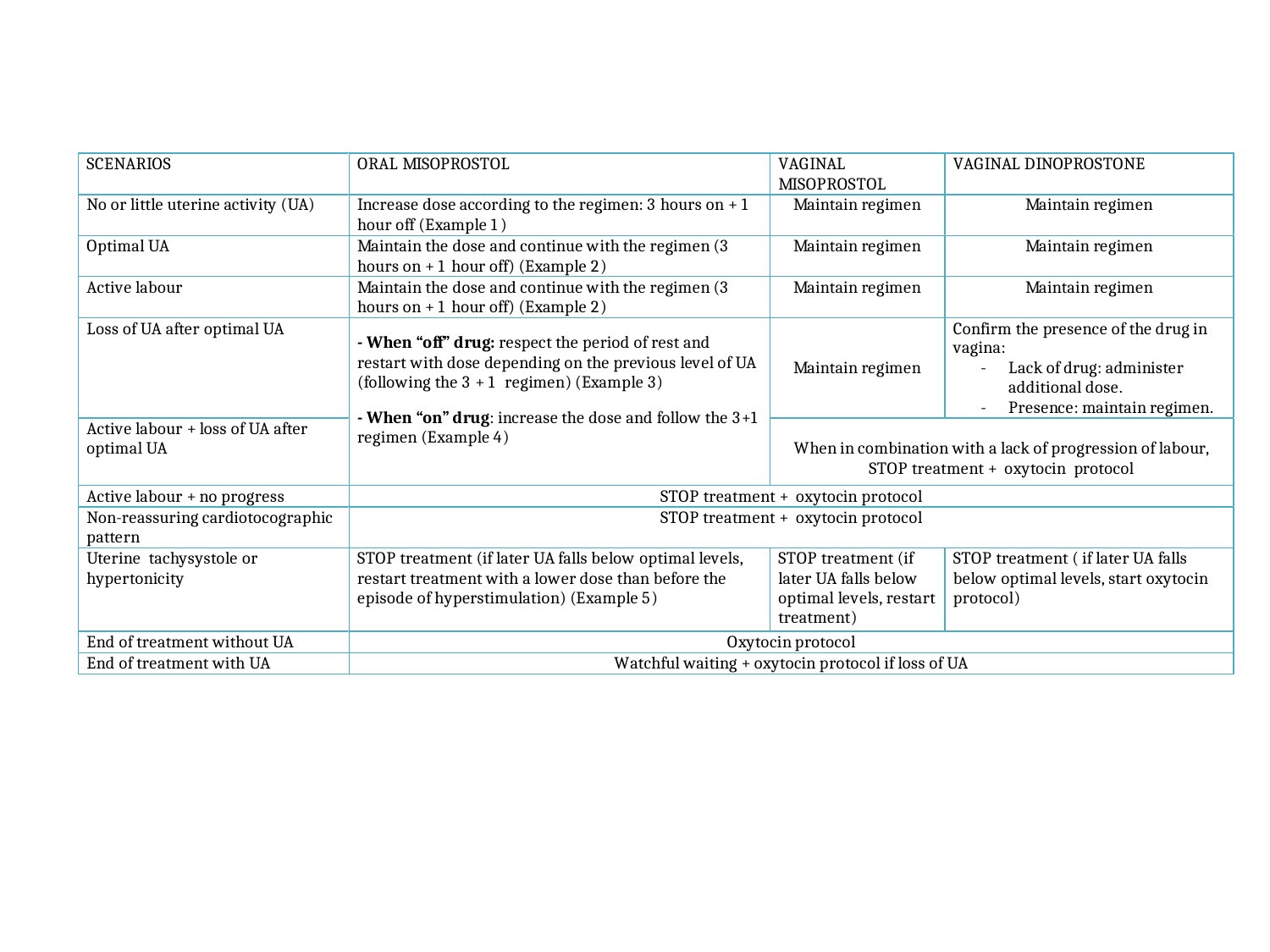

## Slide 2
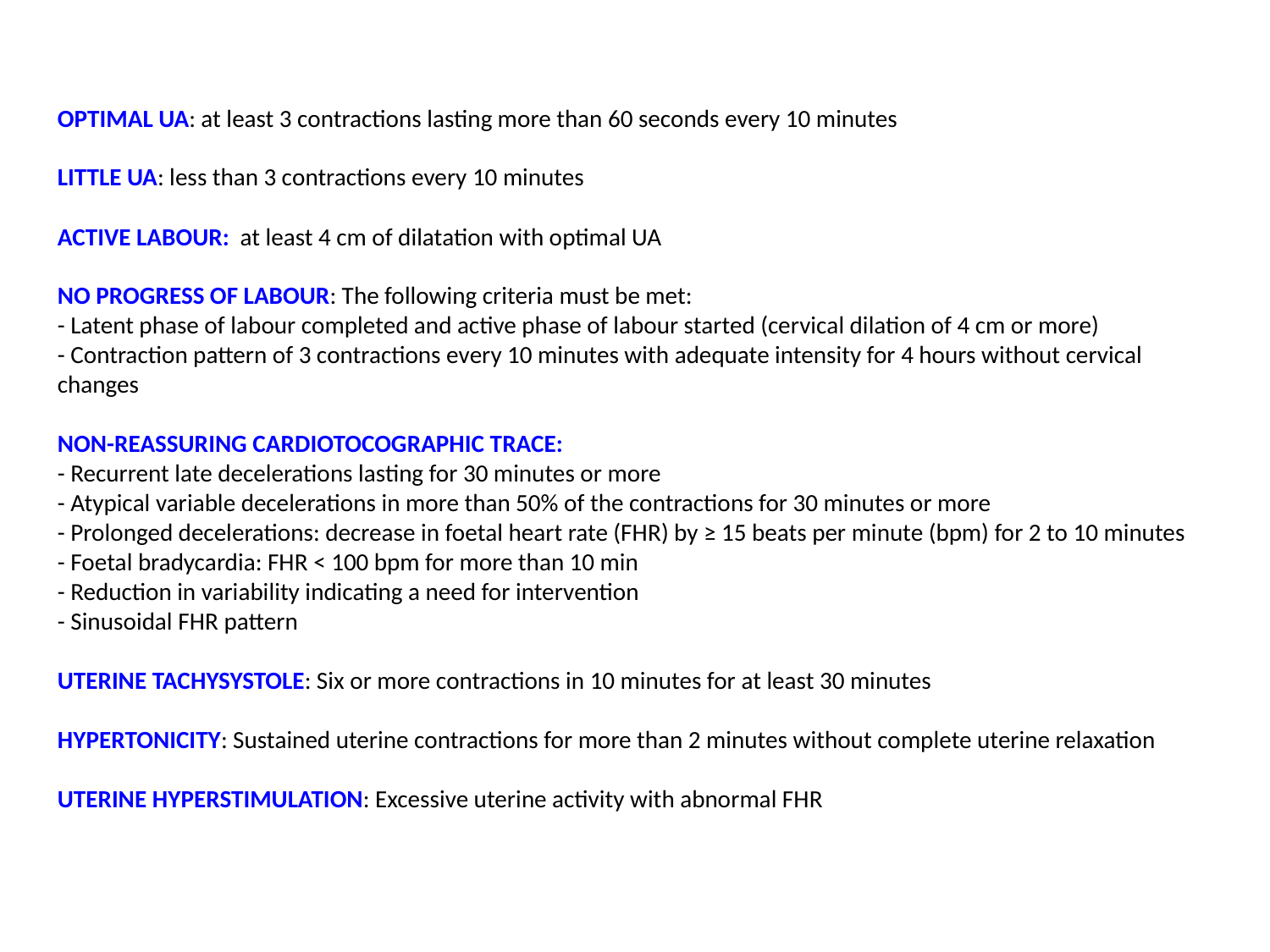

OPTIMAL UA: at least 3 contractions lasting more than 60 seconds every 10 minutes
LITTLE UA: less than 3 contractions every 10 minutes
ACTIVE LABOUR: at least 4 cm of dilatation with optimal UA
NO PROGRESS OF LABOUR: The following criteria must be met:
- Latent phase of labour completed and active phase of labour started (cervical dilation of 4 cm or more)
- Contraction pattern of 3 contractions every 10 minutes with adequate intensity for 4 hours without cervical changes
NON-REASSURING CARDIOTOCOGRAPHIC TRACE:
- Recurrent late decelerations lasting for 30 minutes or more
- Atypical variable decelerations in more than 50% of the contractions for 30 minutes or more
- Prolonged decelerations: decrease in foetal heart rate (FHR) by ≥ 15 beats per minute (bpm) for 2 to 10 minutes
- Foetal bradycardia: FHR < 100 bpm for more than 10 min
- Reduction in variability indicating a need for intervention
- Sinusoidal FHR pattern
UTERINE TACHYSYSTOLE: Six or more contractions in 10 minutes for at least 30 minutes
HYPERTONICITY: Sustained uterine contractions for more than 2 minutes without complete uterine relaxation
UTERINE HYPERSTIMULATION: Excessive uterine activity with abnormal FHR

## Slide 3
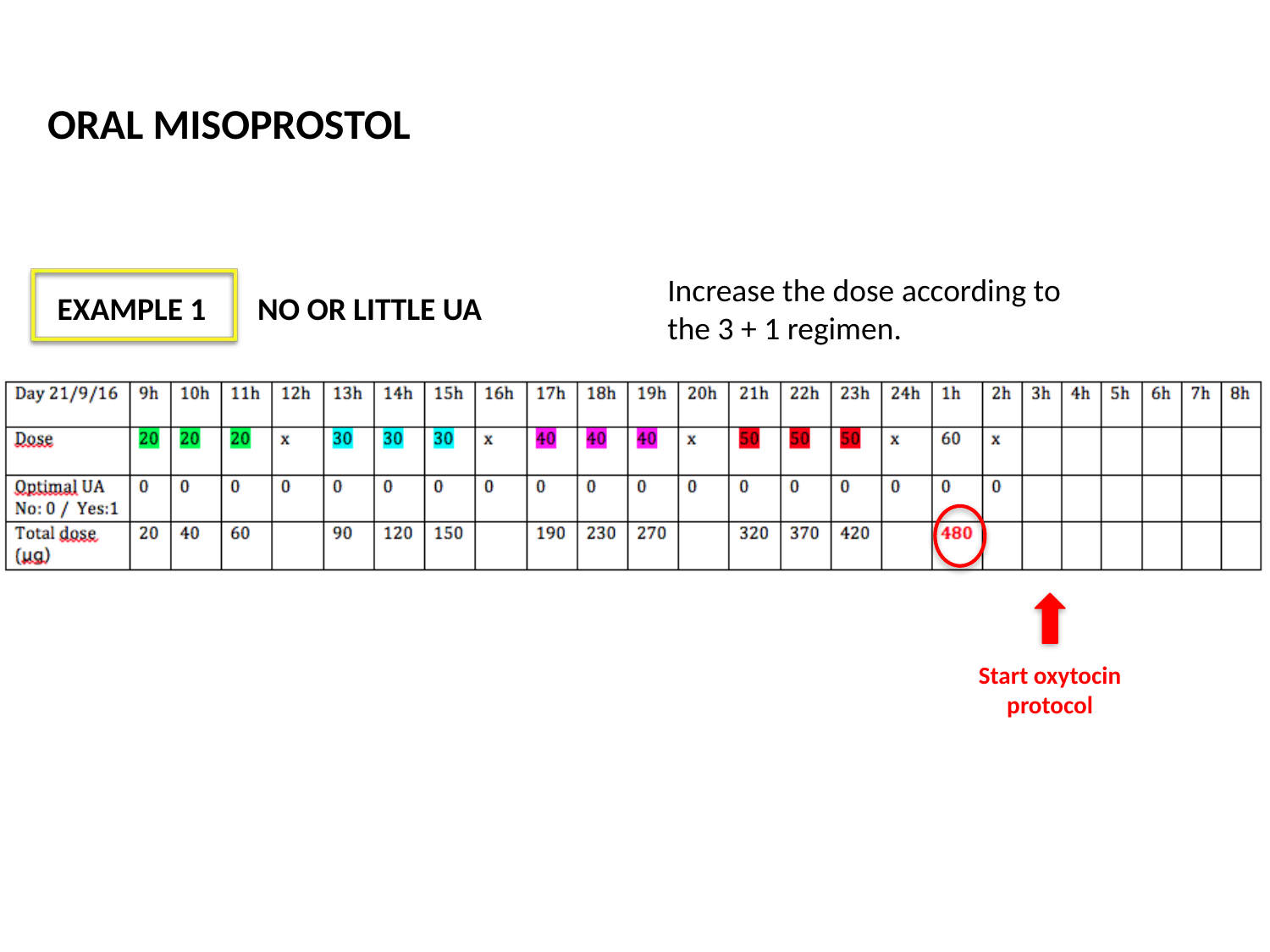

ORAL MISOPROSTOL
Increase the dose according to the 3 + 1 regimen.
EXAMPLE 1
NO OR LITTLE UA
Start oxytocin protocol

## Slide 4
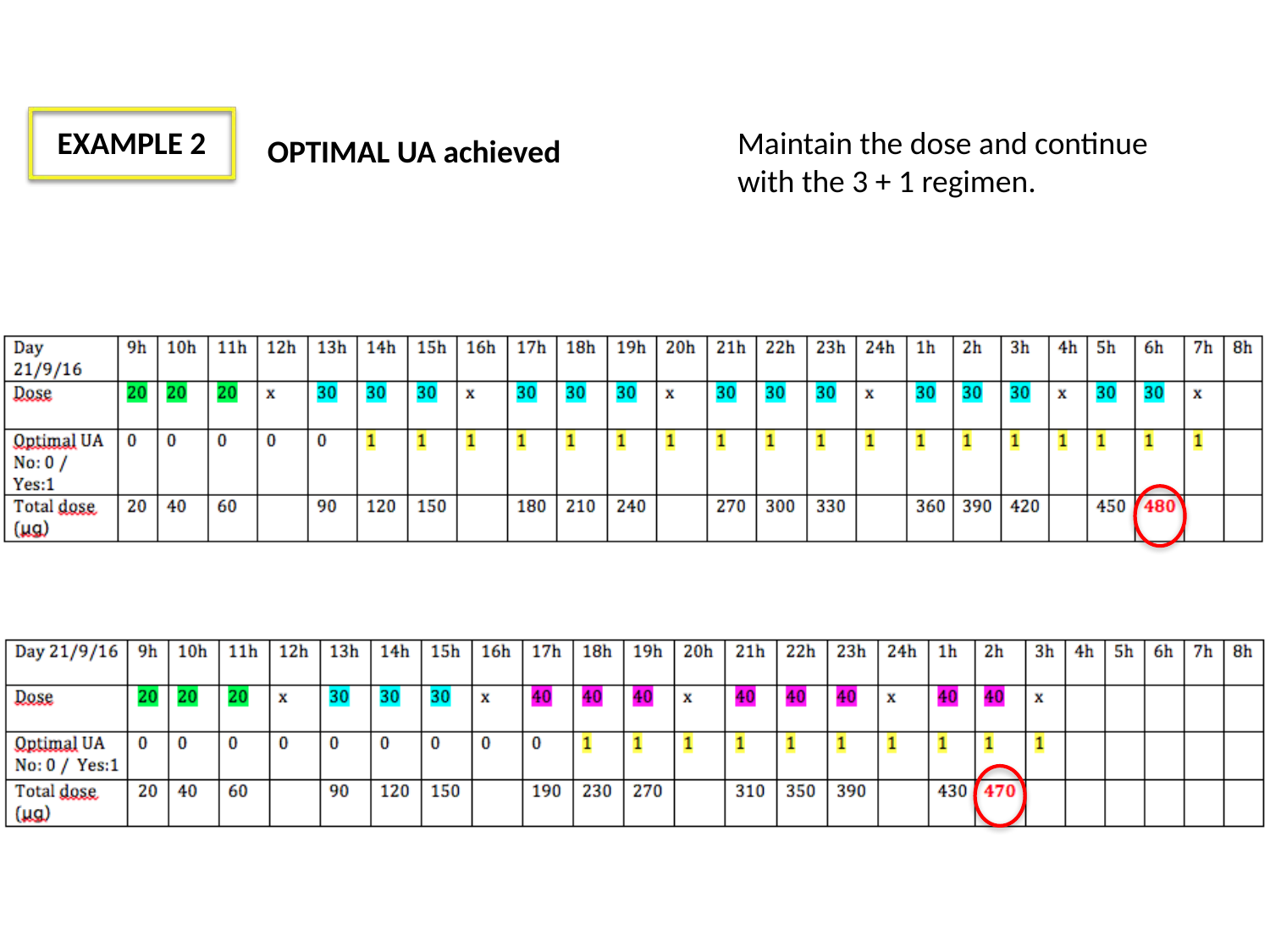

EXAMPLE 2
Maintain the dose and continue with the 3 + 1 regimen.
OPTIMAL UA achieved

## Slide 5
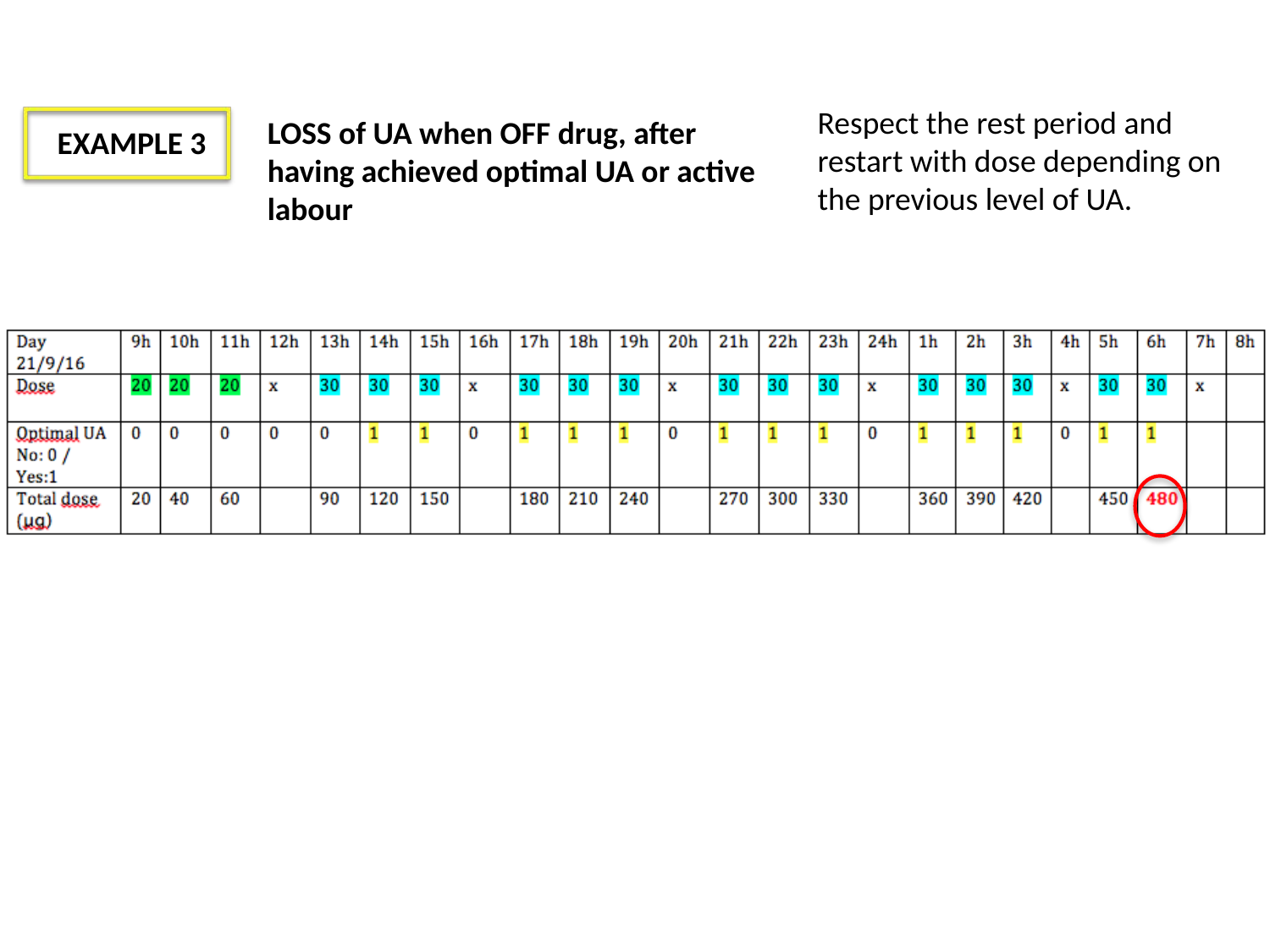

Respect the rest period and restart with dose depending on the previous level of UA.
LOSS of UA when OFF drug, after having achieved optimal UA or active labour
EXAMPLE 3

## Slide 6
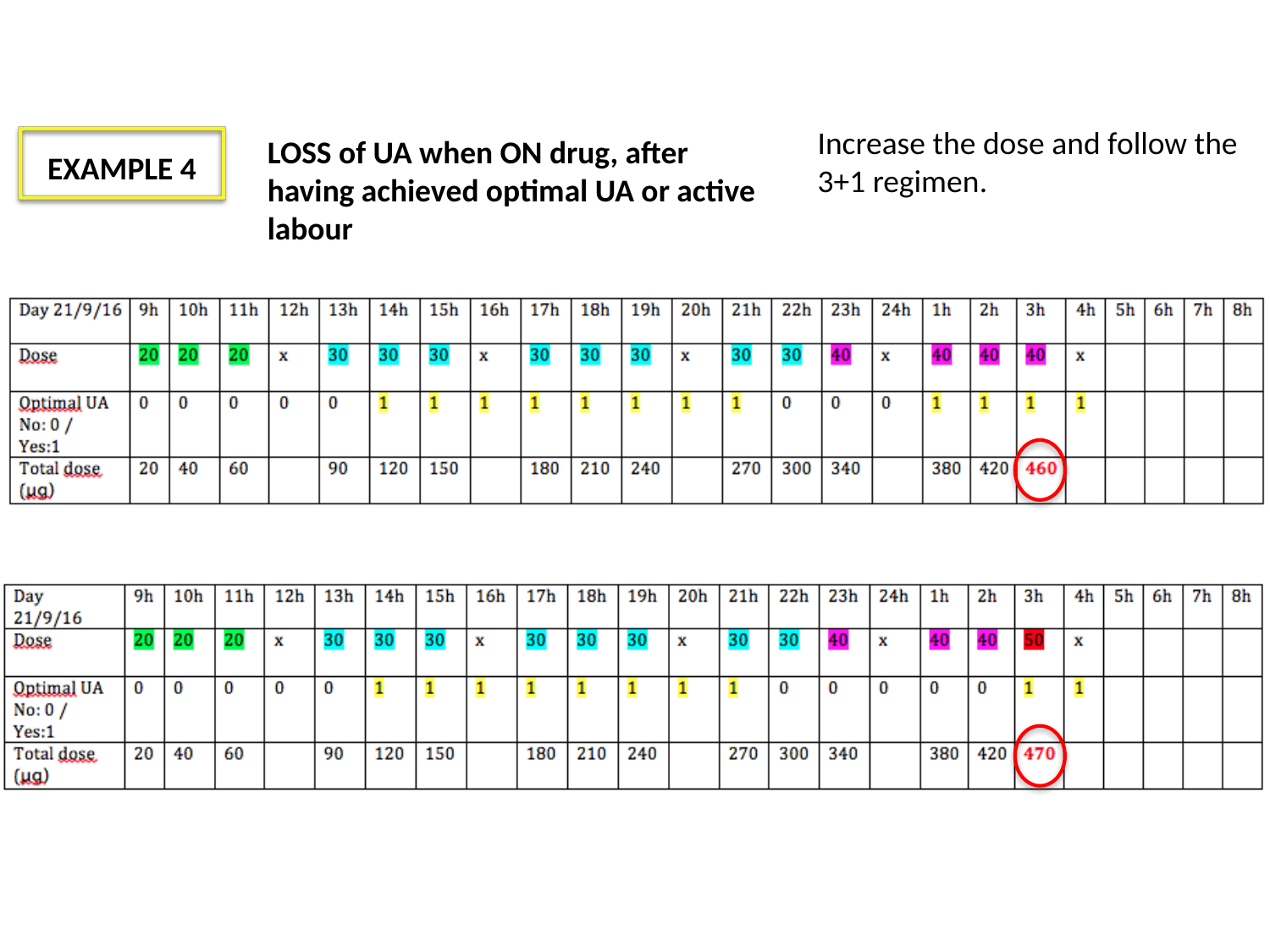

Increase the dose and follow the 3+1 regimen.
LOSS of UA when ON drug, after having achieved optimal UA or active labour
EXAMPLE 4

## Slide 7
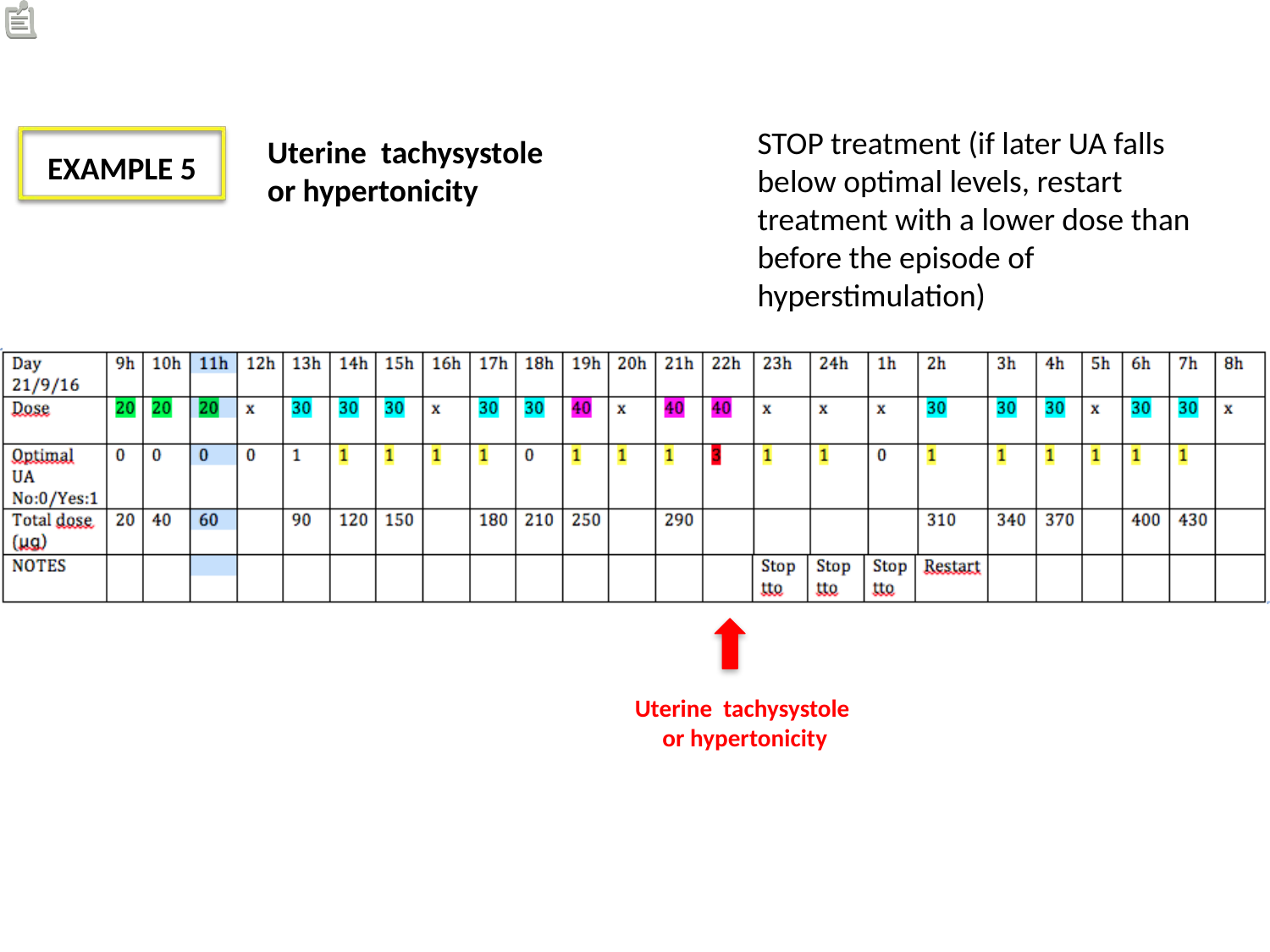

STOP treatment (if later UA falls below optimal levels, restart treatment with a lower dose than before the episode of hyperstimulation)
Uterine tachysystole
or hypertonicity
EXAMPLE 5
Uterine tachysystole
or hypertonicity
